# Phenotyping Neurodisability in Hospital Records in England: A National Birth Cohort Using Linked Administrative Data

**DOI:** 10.1111/ppe.70052

**Published:** 2025-07-25

**Authors:** Ania Zylbersztejn, Philippa Rees, Rashmi D'Souza, Stuart Logan, Ayana Cant, Laura Gimeno, Vincent Nguyen, Jugnoo Rahi, Ruth Gilbert, Katie Harron, HOPE Study, Bianca De Stavola, Lorraine Dearden, Johnny Downs, William Farr, Tamsin Ford, Kate Lewis, Matthew Lilliman, Jacob Matthews, Jennifer Saxton, Isaac Winterburn, Ruth Blackburn, Milagros Ruiz, Matthew Jay, Tony Stone, Farzan Ramzan

**Affiliations:** ^1^ Great Ormond Street Institute of Child Health University College London London UK; ^2^ NIHR Applied Research Collaboration for the Southwest University of Exeter Medical School Exeter UK; ^3^ Centre for Longitudinal Studies, Institute of Education University College London London UK; ^4^ Great Ormond Street Hospital NHS Foundation Trust London UK; ^5^ Institute of Ophthalmology University College London London UK

**Keywords:** administrative data, data linkage, electronic health records, neurodisability, special educational needs

## Abstract

**Background:**

Children with neurodisability often have complex healthcare and educational needs. Evidence from linked administrative health and education data could improve joint working between services.

**Objective:**

To develop a diagnostic code list to identify neurodisability in hospital admission records; to assess the representativeness of this phenotype by characterising children with hospital‐recorded neurodisability and their outcomes.

**Methods:**

We developed a national cohort of singletons born in England between 2003 and 2009, including a nested cohort of children enrolled in primary school, using linked health and education data from the Education and Child Health Insights from Linked Data (ECHILD) database. With expert clinicians, we developed an algorithm based on diagnostic information from hospital records to *phenotype* children with hospital‐recorded neurodisability. We described rates of mortality, planned/unplanned admissions up to 11 years old, and school‐recorded special educational needs (SEN) provision, as proxy measures of the complexity of a child's needs, overall and for over 40 neurodisability subgroups.

**Results:**

Of 3,580,225 children in the birth cohort, 3.6% had hospital‐recorded neurodisability by age 11. The most frequent subgroups included developmental disorders, autism, epilepsy, perinatal brain injury, and cerebral palsy. Children with hospital‐recorded neurodisability had higher mortality and planned/unplanned admission rates compared with their peers, and they accounted for 26% of all planned and 14% of all unplanned hospital admissions before age 11. The nested primary school cohort included 2,956,299 pupils (82.6% of all births), 3.7% of whom had hospital‐recorded neurodisability. 75% of children with hospital‐recorded neurodisability had any school‐recorded SEN provision, and 39% had a record of more intensive provision (compared to 30% and 2.4%, respectively, for their peers).

**Conclusions:**

We derived a phenotype for hospital‐recorded neurodisability, which affects 1 in 28 primary school children in England, with high rates of hospital admissions and SEN provision. This phenotype and its subgroups can be used by service providers and researchers to examine inequalities and inform resource and service provision.

## 1 Background

Neurodisability is an umbrella term for long‐term health conditions associated with impairment of the nervous system, which result in functional limitations (including difficulties with movement, cognition, hearing and vision, communication, emotion, or behaviour) [1]. Examples include autism, learning disability, epilepsy, cerebral palsy, hyperactivity disorders or genetic conditions (such as Down syndrome or inherited metabolic disorders) [1], as well as high‐risk conditions such as extremely preterm birth, perinatal brain injury, or central nervous system (CNS) tumours [2], although a specific diagnosis may not always be defined [1]. Many of these conditions are rare individually (e.g., approximately 0.1% of children have Down syndrome [3], 0.3% have cerebral palsy and other paralytic syndromes [4], 1% of children are autistic [5]), but when grouped together, they include a significant proportion of children (estimates range from 0.2% to 12% depending on data sources and inclusion criteria) [6, 7, 8]. Many children have more than one health condition contributing to neurodisability [1].

Neurodisability captures a heterogenous group of conditions, with varying complexity and severity, but affected children face common challenges. They have disproportionately higher rates of healthcare utilisation, including hospital admissions, emergency department attendances and mental health service contacts than their peers [6, 8, 9, 10, 11, 12, 13]. They are also more likely to require additional support for their learning compared to their unaffected peers [10, 11, 14]. However, population‐level data on the prevalence of childhood neurodisability and long‐term outcomes of children are limited, and most previous research focused on specific conditions or on chronic conditions more broadly. Evidence from whole‐country administrative data can be used to compare service use and provision across the country and inform service planning and joint working between health, education and social care. However, the generalisability of the findings will depend on the representativeness of the population captured in administrative records, which can vary based on how patients interact with healthcare, which sectors are included (e.g., primary or secondary care), and the level of detail in clinical coding [15].

We develop an algorithm, based on diagnostic codes, for *phenotyping* neurodisability in administrative hospital admission records in England and assess the representativeness of this phenotype by characterising children with hospital‐recorded neurodisability. We describe prognostic outcomes for children with hospital‐recorded neurodisability and their unaffected peers in terms of mortality, hospital admission rates and special educational needs (SEN) provision as proxy measures of the complexity of a child's needs. We also compare the estimated prevalence of included subgroups with external evidence and assess the consistency of risk factor–disease associations with clinical expectation.

## 2 Methods

### 2.1 Data Sources

The Education and Child Health Insights from Linked Data (ECHILD) database brings together routinely collected administrative data on health and education in England [16]. Health data comes from Hospital Episode Statistics (HES), a national database covering details of all hospital admissions funded by the National Health Service (NHS) in England. Diagnoses are coded using the International Classification of Diseases version 10 (ICD‐10), and procedures are coded using the Office of Population Censuses and Surveys (OPCS) Classification of Interventions and Procedures. HES are routinely linked to Office for National Statistics (ONS) mortality records, which include dates and ICD‐10 codes for causes of death [17]. National Pupil Database (NPD) captures education records for all children in state‐funded education (including state‐funded specialist provision in mainstream or private schools; approximately 93% of all children are in state‐funded education each year), including information on pupil characteristics, exam results, absences, exclusions, and type of SEN provision [18]. Health and education datasets were linked by NHS England using a multistep deterministic linkage algorithm, described elsewhere [19].

### 2.2 Study Population

We developed a national cohort of singleton children born in England between 01/09/2003 and 31/08/2009 using methods previously described [20, 21]. Children were followed up from birth until death or their 11th birthday. We were not able to account for migration as these data are not available in ECHILD.

We also derived a nested cohort of children who were enrolled in Year 1 (as the first year of compulsory education) at a state‐funded primary school (aged 5/6 years old) to determine the annual and cumulative incidence of SEN provision. From the initial birth cohort, we excluded children who died before the start of Year 1 (i.e., 1st September of the academic year in which they would turn 6 years old), had no linked NPD record, and those who were not enrolled in school in Year 1. Children in the nested school cohort were followed up from Reception (aged 4/5 years, although enrolment in Reception year is not compulsory) to the end of Year 6 (aged 10/11 years).

### 2.3 Phenotyping Hospital‐Recorded Neurodisability

We developed a code list for identifying children with hospital‐recorded neurodisability, which we defined as chronic conditions involving impairment of the brain and/or neuromuscular system and resulting in functional limitations (including difficulties with movement, cognition, hearing, vision, communication, emotion, and behaviour), following a consensus‐based definition from Morris et al. (developed by a multidisciplinary group of health professionals and parents in the UK) [1]. As neurodisability is likely to be under‐recorded in hospital admissions and ECHILD does not include information on functional impairment, we aimed to capture conditions for which more than 50% of affected children would be expected to have neurologic impairment and functional limitations. Given our reliance on diagnostic data, which captures only health needs (without information on functioning or environmental and personal factors), this study necessarily adopts a medical model of disability [22].

We identified a list of candidate conditions by examining existing code lists for phenotyping chronic conditions and selecting a subset of codes involving neurologic impairment (Table S1). We focused on code lists using ICD‐10. We included operative procedure codes specific to neurological conditions (e.g., ventriculoperitoneal shunt for hydrocephalus, Cochlear implant for hearing impairment). Three expert clinicians (PR, RDS, SL) determined whether each of the 1025 candidate ICD‐10 and OPCS codes was expected to be associated with neurologic impairment and functional limitations in more than 50% of children (yes, no, maybe). We retained codes where at least two clinicians answered yes/maybe and excluded codes where at least two clinicians answered “no” (9% of codes) to maximise the sensitivity of the code list. Diagnostic codes were grouped into subcategories (listed in Table 1). We did not include traumatic brain injuries and acquired head injuries since head injury is common, but severity and resulting functional limitations are not well captured in hospital records. The full code list and coding algorithms are available on GitHub (https://github.com/UCL‐CHIG/HOPE_neurodisability) and ECHILD Code List repository (https://code.echild.ac.uk/).

**TABLE 1:** Overview of conditions included in the neurodisability code list.

Neurodevelopmental conditions	Learning disability Developmental disorders Autistic spectrum disorders Hyperkinetic disorders Behavioural (‘conduct’) disorders Tic disorders
Complex neurologic conditions	Cerebral Palsy Epilepsy
Inherited/congenital conditions	Chromosomal anomalies (including Down, Edwards, Patau syndromes) Selected anomalies of sex chromosome (including Klinefelter and Fragile X syndromes) Congenital anomalies of central nervous system (including anencephaly, encephalocele, microcephaly, congenital hydrocephalus, spina bifida) Congenital hypothyroidism Foetal alcohol syndrome Selected inherited metabolic conditions Phakomatoses Other selected high‐risk congenital anomalies
High‐risk conditions affecting brain	Hydrocephalus Paediatric stroke Tumours of brain/central nervous system Inflammatory conditions of brain (including meningitis, encephalitis)
Visual impairment	Bilateral visual impairment Conditions associated with high risk of bilateral visual impairment (e.g., retinopathy of prematurity)
Hearing impairment	Cochlear implant (diagnosis or procedure code) Hearing device Diagnosis of hearing impairment
Impairment of motor function	Degenerative central nervous system (CNS) disorders (including spinal muscular atrophy) Neuromuscular disorders Movement disorders
Perinatal conditions	Severe birth asphyxia Perinatal brain damage (including perinatal stroke, intracranial haemorrhage, hypoxic ischemic encephalopathy, central nervous system infections) Neonatal Abstinence Syndrome Congenital infections (e.g., rubella) Birth weight < 1000 g Gestational age < 27 weeks

Children were indicated to have hospital‐recorded neurodisability if they had a relevant diagnosis or procedure code recorded at any point, aged < 11 years in their hospital admission record, or as any contributory cause of death in their mortality record.

### 2.4 Statistical Analyses

#### 2.4.1 Characterising Children With Hospital‐Recorded Neurodisability

We described the distribution of baseline characteristics recorded in HES birth admission for children with any or no hospital‐recorded neurodisability (year and month of birth, sex, maternal age, birth weight, gestational age). Maternal and birth variables were completed using information from the linked maternal record, where available [23].

The primary outcome was the cumulative incidence of hospital‐recorded neurodisability aged < 1, < 5, and < 11 years old, defined as the number of children with hospital‐recorded neurodisability according to age at first recorded diagnosis divided by person‐years at risk. Person‐years at risk were calculated as the time from birth until first recorded diagnosis, death, or 11th birthday.

Secondary outcomes included proxy measures of the severity or complexity of health or educational needs for children with and without hospital‐recorded neurodisability. These included mean and median length of birth admission, derived using admission and discharge dates from the baby's birth record (although the postnatal care of the mother could cause an extended stay) and planned and unplanned hospital admission rates, defined as the number of admissions per 100 person‐years at risk. Hospital admissions starting on the same day as subsequent discharge (e.g., hospital transfers) were linked together as part of continued hospital care. Admission type (planned/unplanned) was based on the earliest information recorded in an admission. Person‐time at risk was *recalculated* as time from birth to 11th birthday or death, excluding time spent in hospital during admissions. We also derived the proportion of children who died by age 11 years Information about deaths was obtained from linked ONS mortality records and hospital records indicating in‐hospital death. Finally, we report the proportion of children with school‐recorded SEN provision in the nested cohort of children enrolled in primary school. We categorised SEN provision as having a record of an Education, Health and Care Plan (EHCP, a more intensive provision, which may include placement in specialist school settings, arranged and funded by local governments), SEN support (a more common type of provision, arranged and funded by the schools), or no recorded SEN provision [24]. We derived the cumulative proportion of children according to the highest level of SEN provision ever recorded during primary school, using the number of children enrolled in Year 1 (as the first year of compulsory education) as the denominator. We also report the proportion of children with SEN provision in Reception, Year 1, Year 3, Year 6 (corresponding to ages 4/5, 5/6, 7/8 and 10/11 years old, respectively) using the number of children enrolled in a given school year as the denominator.

#### 2.4.2 External Validity of Hospital‐Recorded Neurodisability

Neurodisability captures a broad range of conditions, some of which are likely to be under‐recorded in secondary healthcare settings (e.g., if children receive much of their care from community paediatrics, mental health services or primary care). To assess the representativeness of hospital‐recorded neurodisability, we first compared the cumulative incidence of specific conditions estimated from HES with external references [25]. Second, we assessed the consistency of risk factor–disease associations and prognostic outcomes with clinical expectation [25], by describing the distribution of baseline characteristics (sex, gestational age) and prognostic outcomes (mortality, planned/unplanned hospital admissions, rates of SEN provision) for children with the most common conditions.

For children with any SEN provision, we described school‐recorded reasons for SEN provision, although we note that these data need to be analysed with caution, as categorisation of reasons vary between schools and by pupil demographics (e.g., by ethnic group, gender, for pupils with multiple needs) in which needs are recorded [26, 27]. We estimated the proportion of children with school‐recorded learning disability (grouping “severe learning difficulty” and “profound and multiple learning difficulty”), moderate learning difficulty, speech, language and communication needs, physical disability, visual impairment, hearing impairment and autistic spectrum disorders recorded at any point during primary school. For selected conditions, we used these measures to assess cross‐record concordance [25].

#### 2.4.3 Sensitivity Analyses: Age at First Recording

Neurodisability covers a range of conditions that are likely to be first diagnosed and/or recorded at different ages. We repeated all analyses, separating children with first hospital‐recorded neurodisability before the start of primary school (at age < 5 years) and during primary school (aged 5–10 years old) to explore differences in characteristics according to age at first hospital record.

#### 2.4.4 Missing Data

Of all children, 0.3% had missing sex, 30.6% had missing gestational age, 21.5% had missing birth weight, and 3.3% had missing maternal age. Rates of missing data were comparable between children with and without hospital‐recorded neurodisability. As our aim was to describe covariate distributions to characterise the cohort, without statistical modelling, and outcomes were available for the entire cohort, we did not use multiple imputation. We judged that multiple imputation could introduce unwarranted assumptions about birth characteristics data being missing at random. Instead, missing data were retained as a separate category in descriptive analyses.

### 2.5 Ethics Approval

Ethics approvals for analyses of the ECHILD research database (which this study fits under) were granted by the National Research Ethics Service (17/LO/1494), NHS Health Research Authority Research Ethics Committee (20/EE/0180) and UCL Great Ormond Street Institute of Child Health's Joint Research and Development Office (20PE06/20PE16).

## 3 Results

### 3.1 Characteristics of Children With Hospital‐Recorded Neurodisability

The study cohort included 3,580,225 singleton live births (Figure S1), capturing approximately 96% of singleton births in England (Table S2). Overall, 1.5% had hospital‐recorded neurodisability by age 1, 2.4% by age 5, and 3.6% by age 11. Compared to their peers, children with hospital‐recorded neurodisability were more likely to be male, born at < 37 weeks' gestation, with low birthweight (< 2500 g) or to mothers aged < 20 years old (Table 2). The proportion of children with hospital‐recorded neurodisability increased from 2.9% of those born in 2003–4 to 3.3% in 2008–9, likely due to improvements in coding depth and increased mean number of recorded diagnoses (Table S3) [17].

**TABLE 2:** Cohort characteristics according to having hospital‐recorded neurodisability.

	No hospital‐recorded neurodisability		Any hospital‐recorded neurodisability		Hospital‐recorded neurodisability aged < 5		Hospital‐recorded neurodisability aged 5–10	
	*N*	%	*N*	%	*N*	%	*N*	%
Total								
*Number*	3,452,691		127,534		85,621		41,913	
%			3.6		2.4		1.2	
Sex								
Boys	1,750,108	50.7	78,657	61.7	49,822	58.2	28,835	68.8
Girls	1,692,041	49.0	48,744	38.2	35,666	41.7	13,078	31.2
Missing	10,542	0.3	133	0.1	133	0.2	0	0
Month of birth								
Sep–Oct	591,870	17.1	20,983	16.5	14,233	16.6	6750	16.1
Nov–Dec	560,752	16.2	20,169	15.8	13,710	16.0	6459	15.4
Jan–Feb	548,145	15.9	20,149	15.8	13,456	15.7	6693	16.0
Mar–Apr	565,919	16.4	20,990	16.5	14,184	16.6	6806	16.2
May–Jun	582,671	16.9	22,001	17.3	14,745	17.2	7256	17.3
Jul–Aug	603,334	17.5	23,242	18.2	15,293	17.9	7949	19.0
Year of birth								
2003–04	546,065	15.8	18,449	14.5	12,751	14.9	5698	13.6
2004–05	558,261	16.2	19,325	15.2	13,107	15.3	6218	14.8
2005–06	568,017	16.5	20,505	16.1	13,669	16.0	6836	16.3
2006–07	576,846	16.7	21,573	16.9	14,224	16.6	7349	17.5
2007–08	602,738	17.5	23,125	18.1	15,331	17.9	7794	18.6
2008–09	600,764	17.4	24,557	19.3	16,539	19.3	8018	19.1
Gestational age (weeks)								
23–26	369	0.01	4122	3.2	4122	4.8	NA	NA
27–31	11,879	0.3	5594	4.4	5214	6.1	380	0.91
32–36	117,742	3.4	8235	6.5	5949	6.9	2286	5.5
37–40	1,679,490	48.6	52,169	40.9	32,072	37.5	20,097	47.9
≥ 41	586,339	17.0	16,379	12.8	9980	11.7	6399	15.3
Missing	1,056,872	30.6	41,035	32.2	28,284	33.0	12,751	30.4
Birthweight (g)								
< 1000	1374	0.04	8453	6.6	8450	9.9	< 10	^a^
1000–2499	146,373	4.2	14,002	11.0	10,910	12.7	3100	7.4
2500–3499	1,468,811	42.5	47,061	36.9	29,354	34.3	17,707	42.2
3500–4499	1,051,031	30.4	27,775	21.8	16,214	18.9	11,561	27.6
≥ 4500	43,604	1.3	1458	1.1	909	1.1	549	1.3
Missing	741,498	21.5	28,785	22.6	19,790	23.1	8995	21.5
Maternal age (years)								
< 20	222,271	6.4	10,271	8.1	6508	7.6	3763	9.0
20–24	636,537	18.4	26,423	20.7	16,752	19.6	9671	23.1
25–29	871,784	25.2	30,821	24.2	20,273	23.7	10,548	25.2
30–34	936,720	27.1	29,260	22.9	19,943	23.3	9317	22.2
35–39	531,550	15.4	18,301	14.3	12,781	14.9	5520	13.2
Missing	113,309	3.3	4837	3.8	3547	4.1	1290	3.1

*Note:* NA: not applicable (all children with gestational age < 26 weeks were considered to have hospital‐recorded neurodisability at birth). Counts < 10 were suppressed.

^a^ Indicates suppression of numbers derived from counts < 10.

Children with hospital‐recorded neurodisability had 1,294,401 planned and 414,084 unplanned hospital admissions. They had 10 times higher rates of planned and 4.8 times higher rates of unplanned admissions compared to their peers, and they accounted for 26% of all planned and 14% of all unplanned hospital admissions before age 11 (Table 3). Patterns of planned and unplanned admission rates by year of age were similar for children with and without hospital‐recorded neurodisability (Figure S2). 6.1% (7769) of children with hospital‐recorded neurodisability died before their 11th birthday, compared to 0.3% (11,274) of their peers.

**TABLE 3:** Overview of prognostic outcomes used as proxy measures of the severity or complexity of health or educational needs for children with and without hospital‐recorded neurodisability.

	No hospital‐recorded neurodisability, *N* = 3,452,691 (96.4% of all)	Any hospital‐recorded neurodisability, *N* = 127,534 (3.6% of all)	Hospital‐recorded neurodisability, aged < 5, *N* = 85,621 (2.4% of all)	Hospital‐recorded neurodisability, aged 5–10, *N* = 41,913 (1.2% of all)
Mortality by age at death (years; *n*, %)				
0–10	11,274 (0.3)	7769 (6.1)	7444 (8.7)	325 (0.8)
< 1	9711 (0.3)	5475 (4.3)	5475 (6.4)	Not applicable^a^
1–4	1131 (0.03)	1347 (1.1)	1347 (1.6)	
5–10	432 (0.01)	947 (0.7)	622 (0.7)	325 (0.8)
Length of birth admission (days)				
Mean (SD)	2.5 (5.9)	11.0 (26.8)	14.8 (31.7)	4.4 (12.6)
Median (IQR)	1 (1, 3)	2 (1, 6)	3 (1, 11)	2 (1, 3)
Planned admission rate per 100 person‐years by age (in years)				
< 11				
Rate	3.42	34.24	36.35	30.25
Rate ratio	Reference	10.01	10.63	8.85
< 1	3.66	35.77	47.95	12.55
1–4	3.67	36.77	47.27	16.97
5–10	3.21	32.28	27.05	42.10
Unplanned admission rate per 100 person‐years by age (in years)				
< 11				
Rate	6.48	31.27	35.34	23.61
Rate ratio	Reference	4.83	5.45	3.64
< 1	23.39	87.66	106.88	51.05
1–4	7.74	37.11	46.79	18.88
5–10	2.82	17.85	15.53	22.21
Planned admissions				
Number of admissions	1,747,715	1,294,401	453,314	314,404
Percent of all admissions	74	26	18	8
Unplanned admissions				
Number of admissions	2,453,074	414,084	305,645	108,439
Percent of all admissions	86	14	11	4
SEN provision during primary school (for children with linked education record enrolled in primary school)^b^				
Number of pupils in primary school cohort	2,845,554	110,745	70,797	39,948
Any SEN provision				
Number of pupils	861,349	83,586	50,113	33,473
Percent within each column	30.3	75.5	70.8	83.8
Percent of all with provision	91	9	5	4
SEN support				
Number of pupils	848,754	61,107	33,072	28,035
Percent within each column	29.8	55.2	46.7	70.2
Percent of all with SEN support	93	7	4	3
EHCP				
Number of pupils	67,899	43,717	27,032	16,685
Percent within each column	2.4	39.5	38.2	41.8
Percent of all with EHCPs	61	39	24	15

Abbreviations: EHCP, education, health and care plan; IQR, interquartile range; SD, standard deviation; SEN, special educational needs.

^a^ Children had to be alive aged ≥ 5 to have a diagnosis recorded aged 5–10 years old in hospital records.

^b^ Children could have both SEN support and EHCP (SEN support is usually provided prior to EHCP).

**FIGURE 1:**
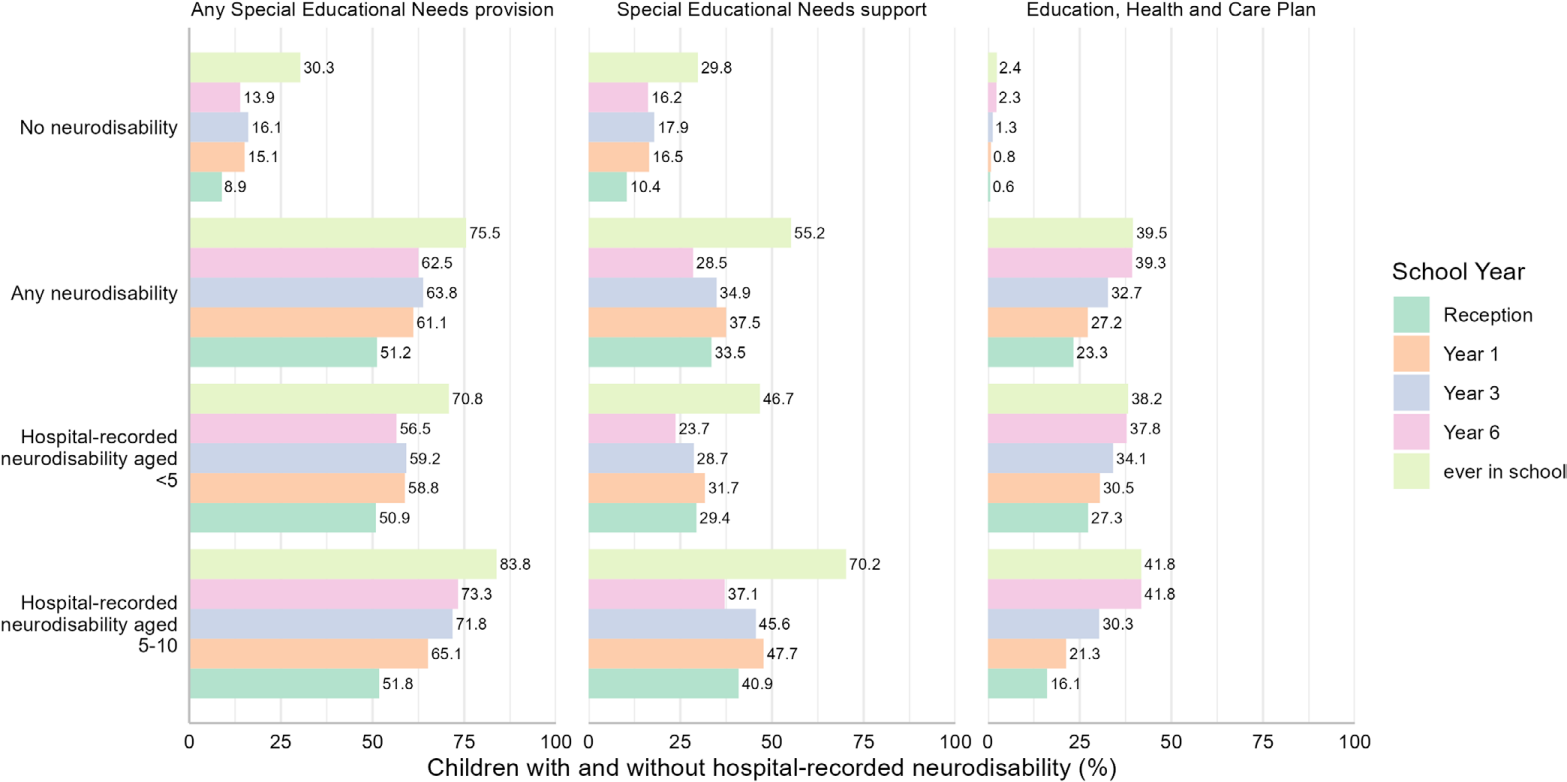
Overview of SEN provision during primary school for children with and without hospital‐recorded neurodisability (by SEN provision type and age at first neurodisability record).* Note that denominators for each year's percentages differ: in Reception‐Year 6 it is the total number of children enrolled in a given school year (which can change between academic years), while for every recorded Special Educational Needs (SEN) it is the proportion of children in the primary school cohort. *Children could have both SEN support and Education, Health and Care Plans—these categories are not overlapping and do not add up to a total of children with any SEN provision.

The nested cohort of primary school children captured 2,956,299 pupils (82.6% of births, Figure S3); 3.7% (110,745) of these had hospital‐recorded neurodisability. Of children with neurodisability, 75% had any SEN provision, 55% had SEN support, and 39% had an EHCP recorded ever during primary school, compared to 30%, 30%, and 2.4%, respectively, for their peers (Figure 1). The proportion of children with an EHCP increased from Reception through Years 1–6 for all children. SEN support peaked in Year 1 and decreased in Years 3 and 6 for children with neurodisability (likely reflecting a move to EHCPs for some children), compared to a peak in Year 3 for children with no neurodisability. Overall, children with a hospital‐recorded neurodisability accounted for 9% of children with any SEN provision, 7% of children with ever recorded SEN support, and 39% of children with an EHCP (Table 2).

### 3.2 External Validity of Hospital‐Recorded Neurodisability

**FIGURE 2:**
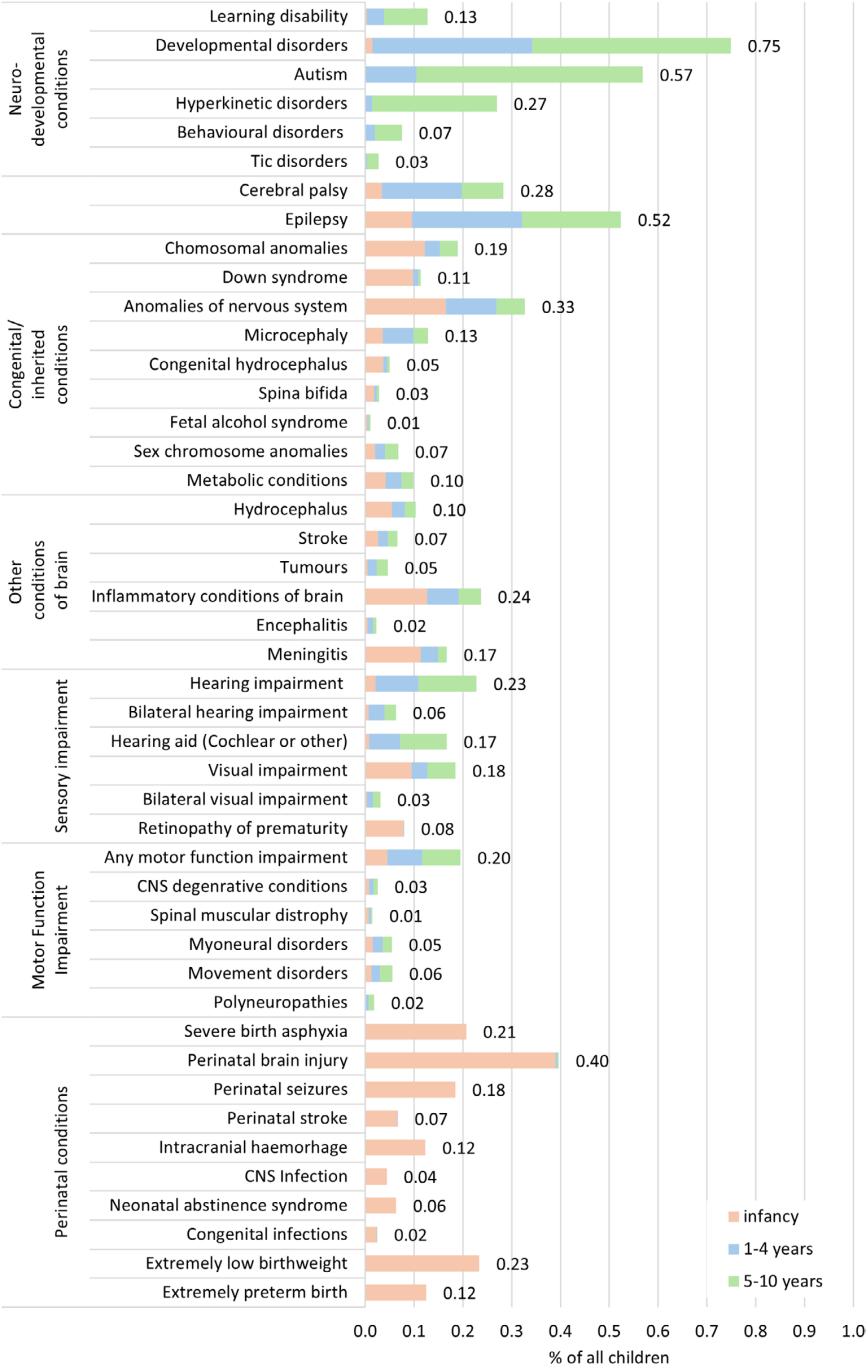
Cumulative incidence of specific neurodisability subgroups by age at first recorded diagnosis.

Of children with hospital‐recorded neurodisability, 40% had a neurodevelopmental condition (most commonly developmental disorders and autistic spectrum disorders) and 15% had epilepsy. One in four children had congenital/inherited conditions (most commonly microcephaly and Down syndrome) and perinatal conditions (most commonly perinatal brain injury, severe birth asphyxia and extremely low birth weight, Figure 2).

**FIGURE 3:**
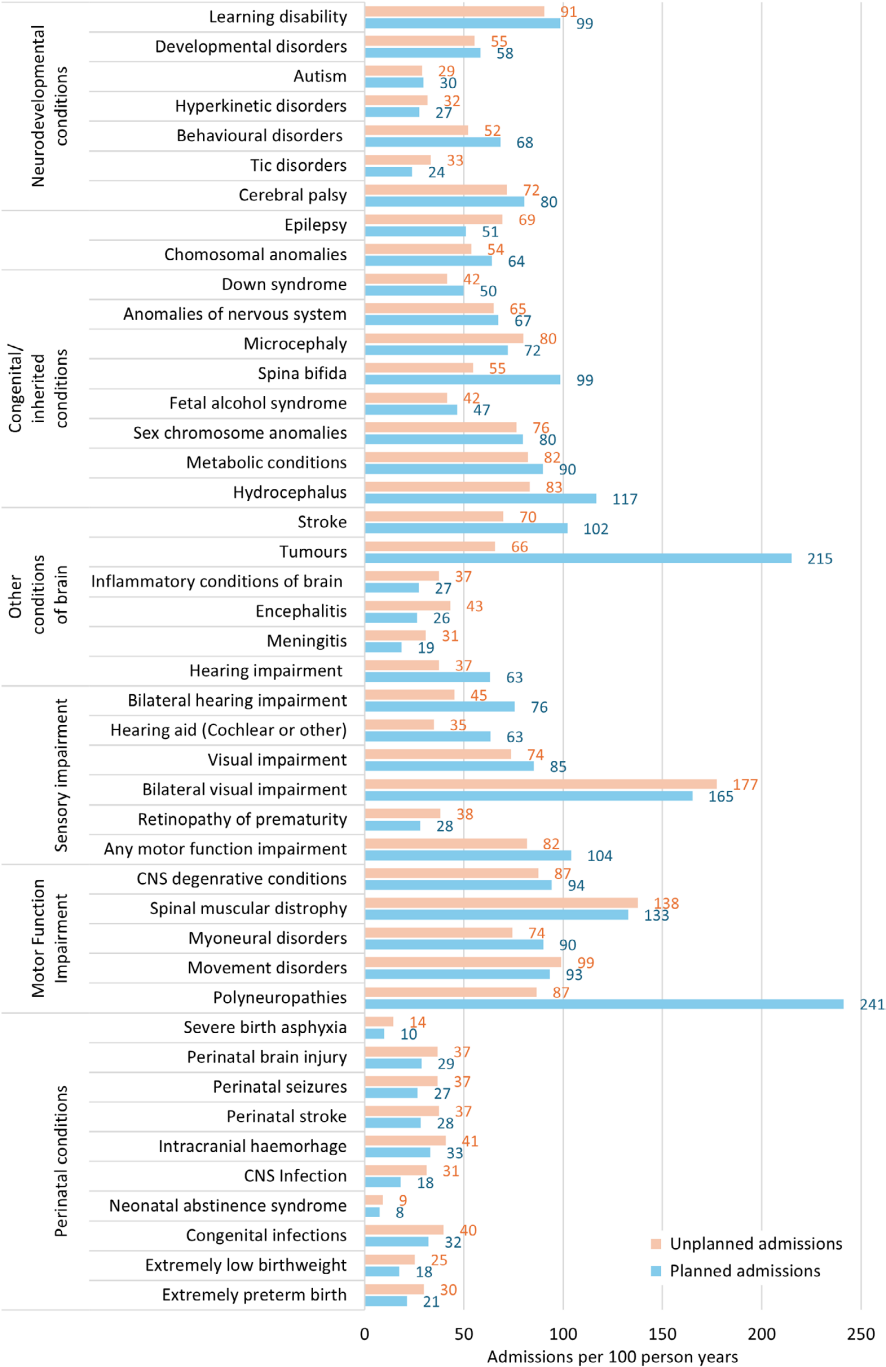
Planned and unplanned hospital admission rates by specific neurodisability phenotype subgroups. CNS, central nervous system.

**FIGURE 4:**
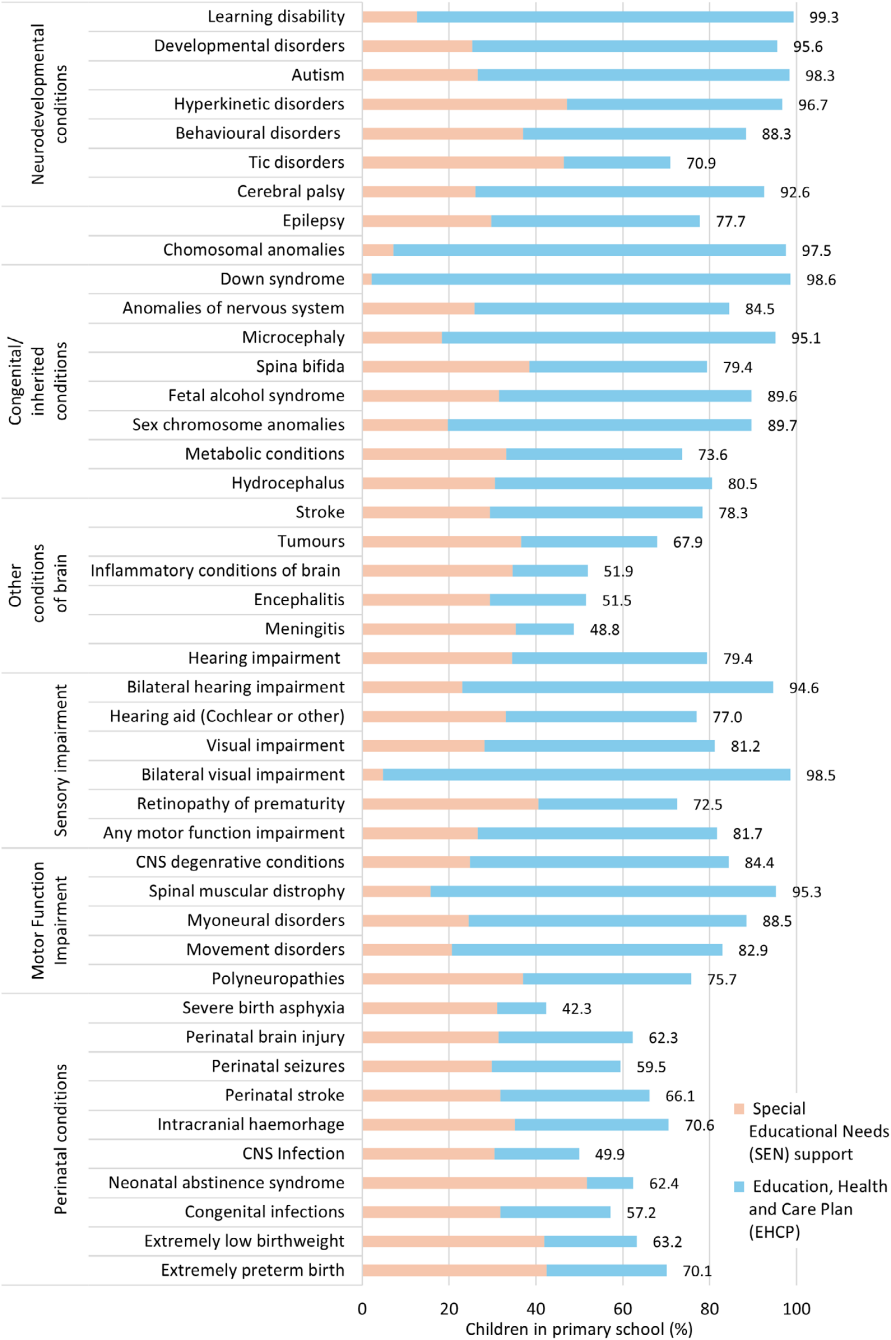
Rates of the highest level of recorded SEN provision (ever during primary school).* CNS, Central nervous system; *SEN provision indicator was hierarchical: Ever EHCP, ever SEN support (and no EHCP), no provision.

Neurodevelopmental conditions (such as autism or learning disability) were substantially under‐recorded in HES compared to population prevalence estimates. Recording of cerebral palsy and epilepsy was comparable to external references (0.3% and 0.5% respectively, Table S4). Children with neurodevelopmental conditions, epilepsy or cerebral palsy had high levels of educational needs (70%–99% received any SEN provision) and healthcare needs (Figures 3 and 4). Similarly, vision and hearing impairment were substantially under‐recorded in HES (Table S4).

Ascertainment of congenital/inherited conditions varied. Anomalies of the CNS were over‐represented in HES compared to estimates from congenital anomaly registries. This could be partially due to differences in the length of “exposure” window, as half of the cases in HES were first recorded after infancy. Chromosomal anomalies screened for during pregnancy (including Down, Edwards and Patau) had prevalence estimates that were similar to previous studies. Children with congenital/inherited conditions had high levels of healthcare and education needs (Figures 3 and 4).

Some specific perinatal conditions were under‐represented. This broad and large group of children had the most heterogeneous needs. Children with perinatal conditions had higher mortality, but lower rates of planned and unplanned hospital admissions and recorded SEN provision than children with other high‐risk conditions (57% had recorded SEN provision, mostly SEN support).

The distribution of sex and preterm birth was consistent with clinical expectation, with a higher proportion of boys and babies born preterm across most conditions (Figures S4 and S5). For example, boys accounted for over 80% of children with autism or hyperkinetic disorders, but girls accounted for just over half of children with spina bifida. Nearly all children with retinopathy of prematurity and extremely low birth weight who had recorded gestational age were born very preterm (< 32 weeks' gestation). Preterm birth rates were substantially higher among children with cerebral palsy, congenital hydrocephalus, and perinatal brain injuries.

The most common school‐recorded reasons for SEN provision among children with neurodisability were speech, language and communication difficulties (30% of pupils), moderate learning difficulties (25%), autistic spectrum disorder (21%), learning disability (18%) and physical disability (16%; Table S5). Cross‐record validation suggests moderate agreement between health and education records (although we do not consider education data to be the gold standard). For example, 71% of children with hospital‐recorded autistic spectrum disorders and 52% with hospital‐recorded learning disability had indications of these conditions in school records; over half of children with microcephaly and chromosomal anomalies had a school‐recorded learning disability; > 60% of children with spina bifida, spinal muscular dystrophy, myoneural conditions and CNS degenerative conditions had school‐recorded physical disability.

### 3.3 Sensitivity Analysis: Age at First Recording

Two thirds of children with neurodisability had a first hospital record of neurodisability aged < 5 years old (*n* = 85,621, 2.4% of all). Compared with children with first hospital‐recorded neurodisability aged 5–10 years, they were more likely born preterm (17.9% vs. 6.4%, respectively, Table 2), with a low birthweight (22.6% vs. 7.4%). They also had longer birth admissions, higher rates of hospital admissions aged < 5 years old, but lower rates of admissions aged 5–10 years old (Table 3). Children with first hospital‐recorded neurodisability aged 5–10 years had higher rates of SEN provision during primary school (both SEN support and EHCPs, Figure 2).

Of conditions first recorded before age 5, perinatal conditions were the most common (0.86% of all children), followed by congenital/inherited conditions (0.66%), developmental disorders (0.34%), epilepsy (0.32%) and cerebral palsy (0.20%). In contrast, the most common conditions first recorded between 5 and 10 years old were autism (incidence aged 5–10 was 0.46%), followed by developmental disorders (0.41%), hyperkinetic disorders (0.26%), epilepsy (0.20%) and congenital/inherited conditions (0.16%). Most children with neurodevelopmental conditions had their first hospital‐recorded diagnosis during primary school (ranging from 55% for developmental delay to 95% for hyperkinetic disorders).

## 4 Comment

### 4.1 Principal Findings

In this national birth cohort of over 3.5 million singleton children, 3.6% of children had neurodisability or an associated high‐risk condition recorded in hospital records, aged < 11 years old. They had 10 times higher rates of planned and five times higher rates of unplanned hospital admissions than their peers, accounting for one in four planned and one in seven unplanned admissions from birth to age 11. Three‐quarters of children with hospital‐recorded neurodisability received any SEN provision at least once during primary school, half of whom had an EHCP, accounting for one in 10 pupils with any SEN provision and four in 10 pupils with EHCPs. Rates of mortality, hospital admissions, and SEN provision varied across the subgroups of the neurodisability phenotype but were consistently higher than among peers without neurodisability.

### 4.2 Strengths of the Study

This is the first whole population longitudinal cohort study to describe the prevalence and prognostic outcomes of children with neurodisability in England. We used a national birth cohort capturing 96% of singleton births in England. Diagnostic data enabled the identification of hospital‐recorded conditions involving neurodisability. As over 80% of children in the birth cohort in HES were linked to education records and enrolled in state‐funded primary school, we could assess the proportion of children with and without hospital‐recorded neurodisability, who were assigned SEN provision—an indication of additional learning needs not available in HES data. ECHILD's large sample size and longitudinal data collection for all patients in the NHS and state‐funded education in England enabled us to further stratify our analyses of health and education outcomes by subgroups of neurodisability, offering a unique resource for studying long‐term outcomes of children with rare conditions and their peers.

### 4.3 Limitations of the Data

We were limited to clinical information recorded in hospital admission records, underestimating the true prevalence of neurodisability and high‐risk conditions. Multiple births are associated with a higher risk of neurodisability, but were not included due to an increased risk of false matches. Many children with neurodisability receive much of their care from community paediatrics, mental health services or primary care, and they could be misclassified as not having neurodisability in our study if they were not admitted to hospital. We therefore join the call for action to support timely linkages of data across different parts of the NHS, especially primary care [28]. Better recording of diagnostic data in HES outpatient records (currently, most diagnoses are missing) could also increase case ascertainment and generalisability of findings.

We found that some conditions (e.g., congenital anomalies) were over‐represented in HES. This could reflect the use of diagnostic codes for suspected/unconfirmed cases. High prevalence of congenital anomalies recorded in hospital has also been reported using data from Scotland and Sweden [29, 30]. We also observed increases in the prevalence of hospital‐recorded neurodisability over time, likely reflecting the introduction of a pay‐for‐performance reimbursement system, which led to improved coding depth [17]. Linkage to the National Congenital Anomaly and Rare Disease Registration Service (NCARDRS) and gold standard datasets for other conditions is needed for validation of the specificity and sensitivity of ICD‐10 codes in hospital records [31]. Alternatively, validation of ICD‐10 codes can be carried out via case note review [32, 33], for which we did not have permissions.

Finally, our definition of neurodisability followed a medical model of disability, as we focused on clinical information recorded in hospitals to capture health needs, but we did not have reliable indicators of functioning or environmental and personal factors [22]. Not all children with a hospital‐recorded diagnosis of neurodisability will have functional impairment [33]. Our definition of neurodisability included a broad range of subgroups capturing conditions considered by expert clinicians to be more than 50% likely to involve neurodisability. Our finding that 75% of children with the neurodisability phenotype were assigned SEN provision confirms a high prevalence of cognitive or functional impairment, although this varied between different groups of conditions. Proxy measures of the complexity of needs available from linked health and education data are quite limited. More in‐depth recording of children's needs (including speech, language and communication needs, sensory impairment, mental health and behavioural needs, mobility issues or technology dependency) across linked primary care records, mental health services and community child health and child and adult social services would enable population‐level studies to quantify children's needs, making them visible to policy makers and services [34, 35].

### 4.4 Interpretation

Our study adds new evidence to limited research on the prevalence of neurodisability in England. We found that 3.6% of singleton children had hospital‐recorded neurodisability by age 11 years These children account for a disproportionately high proportion of both hospital admissions and SEN provision. Further research will describe the outcomes of children with neurodisability across primary school, into adolescence and early adulthood and examine the fairness and effectiveness of SEN provision for children with neurodisability in England [24, 36, 37, 38].

Our methods form a valuable resource for population data science studies aiming to describe outcomes for children with neurodisability, although the complexity and additional health and educational needs depend on age. For example, children with hospital‐recorded neurodisability before primary school were more likely to have perinatal conditions or congenital/inherited conditions, while those with neurodisability first recorded during primary school were more likely to have neurodevelopmental conditions. We therefore recommend that analyses using this diagnostic code list always include a subgroup analysis to understand the characteristics of the captured population and consider the conditions that were especially under‐represented to inform interpretation of findings.

## 5 Conclusions

Children with hospital‐recorded neurodisability are a small group with much higher rates of mortality, secondary healthcare utilisation and need for educational support than their peers. This code list for neurodisability can be used by researchers, healthcare services and the education sector to monitor variation in the prevalence of neurodisability across disadvantaged groups and to inform resource and service allocation. Better population‐level data are needed, however, to make the needs of children with neurodisability visible. This requires linkage of data across different services that these children interact with, as well as consistent coding of their needs, including measures of functional impairment that are relevant to additional support from education and social care as well as from healthcare.

## Author Contributions

The study was designed by A.Z., R.G. and K.H. A.Z. carried out literature review to develop the code list, P.R., R.D.S., T.S. and J.R. provided clinical input on the code list. V.N., A.C. and L.G. supported data management. A.Z. developed the cohort, analysed the data and wrote the first draft of the manuscript. All authors interpreted the data and contributed to subsequent drafts of the manuscript. All authors have seen and approved the final version.

## Ethics Statement

Permissions to use linked, de‐identified data from Hospital Episode Statistics and the National Public Database were granted by DfE (DR200604.02B; 05/08/2020) and NHS Digital (DARS‐NIC‐381972‐Q5F0V‐v0.5; 03/09/2020). Ethical approval for the ECHILD project was granted by the National Research Ethics Service (17/LO/1494), NHS Health Research Authority Research Ethics Committee (20/EE/0180) and UCL Great Ormond Street Institute of Child Health's Joint Research and Development Office (20PE06/20PE16).

## Consent

Consent from patients is not required here.

## Conflicts of Interest

The authors declare no conflicts of interest.

## Supporting information


Data S1.

## Data Availability

The authors have nothing to report. ECHILD data are not shared publicly in line with data‐sharing agreements with NHS Digital and the Department for Education. ECHILD can be accessed by accredited researchers through application via the ECHILD team (www.echild.ac.uk) and the ONS Research Accreditation Panel.
