# Supplementary material for: Phenotyping Neurodisability in Hospital Records in England: A National Birth Cohort Using Linked Administrative Data
Source: Paediatr Perinat Epidemiol. 2025 Jul 25;39(8):680–94. doi: 10.1111/ppe.70052 (PMC12658309; doi:10.1111/ppe.70052)
Supplement: Supplementary file 1 — Data S1. [file PPE-39-680-s001.docx]

# Supplementary materials

Appendix Table 1 – overview of code lists used to develop an initial list of ICD-10 codes for review by expert clinicians.*

| Paper | Definition of chronic condition | Codes considered in the initial code list for expert review |
| --- | --- | --- |
| Cohen et al. *“Patterns and costs of health care use of children with medical complexity”*^1^ | This code list focused on children with medical complexity, defined as children who have complex underlying chronic health conditions that are typically associated with significant functional status limitations. This includes children with neurologic impairment (NI) and medical technology assistance (TA). NI included diagnoses consistent with static or progressive neurologic, genetic, or other disease that typically results in either functional and/or intellectual impairment. | We included all codes indicating neurologic impairment for initial review |
| Thompson J, et al. *“Identification of Children With High-Intensity Neurological Impairment*”^2^ | This code list focused on high-intensity neurologic impairment, which included neurological diagnoses that are expected to last 12 months (unless death) or longer and result in significant functional impairments requiring subspecialty medical care.^2^ This builds on a code list developed by Berry et al. using ICD-9 codes.^3^ | We included all codes for initial review |
| Hardelid P et al. “*Estimating the prevalence of chronic conditions in children who die in England, Scotland and Wales: a data linkage cohort study*.”^4^ | This chronic conditions’ code list was developed for use in mortality records and hospital admissions records from NHS Hospitals in England. Chronic conditions were defined as any health problem likely to require follow-up for more than 1 year, where follow-up could be repeated hospital admission, specialist follow-up through outpatient department visits, medication or use of support services. | We included codes in the *neurological* sub-category for initial review although not all conditions are associated with functional impairment.  We also noted that some associated conditions were included in other sub-categories (e.g. in mental health or metabolic categories). |
| Feudtner C et al. “*Pediatric complex chronic conditions classification system version 2: Updated for ICD-10 and complex medical technology dependence and transplantation*.”^5^ | This code list focussed on complex chronic conditions, defined as medical conditions that can be reasonably expected to last at least 12 months (unless death intervenes) and involve either several different organ systems or 1 organ system severely enough to require specialty pediatric care and probably some period of hospitalization in a tertiary care centre. | We included all neurologic and neuromuscular conditions for initial review, although not all conditions are associated with functional impairment.  We also noted that some associated conditions were included in other sub-categories (e.g. chromosomal anomalies, birth asphyxia, cerebral haemorrhage) |
| Fraser L. et al, “*Estimating the current and future prevalence of life-limiting conditions in children in England*”^6^ | This code list focussed on life-limiting conditions (for which there is no reasonable hope of cure and from which children or young people will die) and life-threatening conditions (for which curative treatment may be feasible but can fail, such as cancer) | We included all conditions included in the “Neurology & Oncology: Central Nervous System” subgroup, although some conditions associated with neurodisability were also included in other categories. |
| Jarvis S et al. “*The impact of neurological disorders on hospital admissions for children and young people: a routine health data study*”^7^ | This code list included all children with a neurological diagnosis who, depending on the severity of their condition, may need to be seen by a paediatric neurologist (e.g. epilepsy, cerebral palsy, Duchenne muscular dystrophy, Batten disease). | We used all codes in this code list for initial review, although not all neurologic conditions are associated with functional impairment. |
| Sheehan et al. “*Recording of intellectual disability in general hospitals in England 2006–2019: Cohort study using linked datasets*”^8^ | This code list was developed to indicate adults with learning disability in hospital admission records. | We used all codes included in this code list for initial review, although they focus on adult population. |
| Zylbersztejn A et al. *“Trends in hospital admissions during transition from paediatric to adult services for young people with learning disabilities or autism: population-based cohort study”*^9^ | This code list was developed to indicate hospitalised young people with learning disability, including conditions associated with learning disability in more than 30% of cases (to increase case ascertainment in hospital admission records). | We used all codes included in this code list for initial review. |
| Gale et al. “*Neonatal brain injuries in England: population-based incidence derived from routinely recorded clinical data held in the National Neonatal Research Database*”^10^ | This paper sets out a definition of neonatal brain injury as brain injury occurring at or soon after birth. It sets out conditions to be included and other conditions affecting brain around the time of birth that should be excluded. | We translated all conditions into ICD-10 codes, by reviewing ICD-10 codes and by examining codes included in a composite neonatal adverse outcome indicator developed for English hospital admissions data.^11^ |
| Barsch Bergqvist et al “*Term-born infants with a perinatal stroke frequently had seizures and were prescribed anti-seizure medication at discharge*”^12^ | The code list included ICD-10 codes for perinatal stroke | We used all codes included in this code list for initial review. |
| Milan et al. “*The ASPECT Hydrocephalus System: a non-hierarchical descriptive system for clinical use*”^13^ | This paper listed ICD-10 codes for hydrocephalus. | We used all codes included in this code list for initial review. |
| Carter et al. *“Identifying cerebral palsy from routinely-collected data in England and Wales”*^14^ | This paper listed ICD-10 codes for indication of cerebral palsy in hospital admission records in England and Wales. | We used all codes included in this code list for initial review. |
| Guttmann et al. *“Long-term mortality in mothers of infants with neonatal abstinence syndrome: A population-based parallel-cohort study in England and Ontario, Canada”*^15^ | This paper used indication of neonatal abstinence syndrome in baby’s birth record to examine maternal outcomes using linked birth and delivery records. | We used all codes included in this code list for initial review. |

* This is not a comprehensive list of all code lists for paediatric chronic conditions, and other code lists are available. We focused on code lists using ICD-10 codes, which we routinely apply to Hospital Episode Statistics (HES) data. We did not consider code lists using ICD-9 or ICD-10 Clinical Modification (ICD-10-CM) codes, as these are not available in HES data. Where a code list for a specific condition was identified, we included these codes for initial review.

Appendix Figure 1 – Flow chart describing derivation of the study cohort

Singleton live births in final cohort (n=3,580,225)

Babies with birth admissions to NHS hospital in England between 1st September 2003 and 31st August 2009 (n=3,735,682)

x

Implausible age at admission (n=15,063)

Likely false match (multiple records with conflicting birth information for the same individual, n=1,806)

Likely stillbirth (n=25,092)

Total exclusions^a^ (*n* = 155,457, 4.2%)

Non-English residents (n=5,014)

*NHS: National Health Service;*^a^*All exclusions were defined using criteria published previously*^16^

Appendix Table 2 – Comparison of birth cohort coverage compared to published figures from the Office for National Statistics

|  | **England & Wales (official statistics)** | | | **England** | | **ECHILD birth cohort (births per school year)** | |
| --- | --- | --- | --- | --- | --- | --- | --- |
| **Calendar year** | **All live births**^17,18^ | **Singleton live births**^17,18^ | **% of singleton** | **all live births**^17,18^ | **Estimated number of singleton live births*** | **Singleton live births** | **% of all singleton live births in England** |
| 2004 | 639,721 | 620,824 | 97% | 607,184 | 589,248 | 564,514 | 96% |
| 2005 | 645,835 | 626,862 | 97% | 613,028 | 595,019 | 577,586 | 97% |
| 2006 | 669,601 | 649,421 | 97% | 635,748 | 616,588 | 588,522 | 95% |
| 2007 | 690,013 | 669,170 | 97% | 655,357 | 635,561 | 598,419 | 94% |
| 2008 | 708,711 | 687,087 | 97% | 672,809 | 652,280 | 625,863 | 96% |
| 2009 | 706,248 | 683,471 | 97% | 671,058 | 649,416 | 625,321 | 96% |
| Total | 4,060,129 | 3,936,835 | 97% | 3,855,184 | 3,738,112 | 3,580,225 | 96% |

**estimated under the assumption that the proportion of all births that are singletons is the same in England as in England and Wales*

Appendix Table 3 – Mean and median number of diagnoses recorded per episode of care in HES data for children in the ECHILD birth cohort (born between September 2003 and August 2009) by age at admission and calendar year of admission

| **Calendar year** | **Episodes at <1 year old** | | **Episodes at 1-4 years old** | | **Episodes at 5-10 years old** | |
| --- | --- | --- | --- | --- | --- | --- |
|  | **Mean (SD)** | **Median (IQR)** | **Mean (SD)** | **Median (IQR)** | **Mean (SD)** | **Median (IQR)** |
| 2003 | 1.68 (1.19) | 1 (1-2) | NA | NA | NA | NA |
| 2004 | 1.68 (1.21) | 1 (1-2) | 1.85 (1.26) | 1 (1-2) | NA | NA |
| 2005 | 1.70 (1.26) | 1 (1-2) | 1.88 (1.29) | 2 (1-2) | NA | NA |
| 2006 | 1.74 (1.32) | 1 (1-2) | 1.95 (1.35) | 2 (1-2) | NA | NA |
| 2007 | 1.74 (1.34) | 1 (1-2) | 1.99 (1.39) | 2 (1-2) | NA | NA |
| 2008 | 1.76 (1.39) | 1 (1-2) | 2.00 (1.40) | 2 (1-2) | 1.97 (1.37) | 2 (1-2) |
| 2009 | 1.83 (1.48) | 1 (1-2) | 2.08 (1.46) | 2 (1-2) | 2.04 (1.41) | 2 (1-2) |
| 2010 | 2.10 (1.55) | 2 (1-2) | 2.22 (1.60) | 2 (1-3) | 2.17 (1.55) | 2 (1-3) |
| 2011 | NA | NA | 2.30 (1.72) | 2 (1-3) | 2.26 (1.67) | 2 (1-3) |
| 2012 | NA | NA | 2.35 (1.71) | 2 (1-3) | 2.33 (1.73) | 2 (1-3) |
| 2013 | NA | NA | 2.42 (1.78) | 2 (1-3) | 2.46 (1.87) | 2 (1-3) |
| 2014 | NA | NA | 2.46 (1.83) | 2 (1-3) | 2.51 (1.95) | 2 (1-3) |
| 2015 | NA | NA | NA | NA | 2.55 (2.08) | 2 (1-3) |
| 2016 | NA | NA | NA | NA | 2.63 (2.23) | 2 (1-3) |
| 2017 | NA | NA | NA | NA | 2.77 (2.35) | 2 (1-3) |
| 2018 | NA | NA | NA | NA | 2.97 (2.54) | 2 (1-4) |
| 2019 | NA | NA | NA | NA | 3.14 (2.67) | 2 (1-4) |

*HES: Hospital Episode Statistics, IQR: interquartile range, NA: not applicable (meaning that the there were no admissions at given age for the study cohort), SD: Standard Deviation*

Appendix Figure 2 – Planned and unplanned admission rates for children with and without hospital-recorded neurodisability by age in the ECHILD birth cohort (born between September 2003 and August 2009)

Appendix Figure 3 – Flow chart describing derivation of nested cohort of primary school children

Singleton live births enrolled in primary school (n=2,956,299)

Singleton live births in the study cohort (n=3,580,225)

x

Died before start of primary school (n=17,813)

Not linked to NPD^a^ (n=501,064)

Not enrolled in any primary school^b^ (n=105,049)

Total exclusions (*n* = 623,926, 17.4%)

*NHS: National Health Service; NPD: National Pupil Database*^a^*Children with no linked NPD record could be missed links, they could have migrated out of England before school start, or they could be enrolled in private schools
^b^Children with a linked NPD record but not enrolled in primary school may be attending private school (and only enrol in state-funded school at later age)*

Appendix Table 4 – Comparison of cumulative incidence estimates at ages <1, <5 and <11 years old for selected conditions using ECHILD and evidence from literature on population-level disease freqyency (orange indicates under-reporting, blue indicates over-estimate, green indicates good estimate and grey indicated lack of comparable data)

| **Condition** | **Cumulative incidence from HES by age at recorded diagnosis** | | | **Evidence from literature** |
| --- | --- | --- | --- | --- |
|  | **<1yr** | **<5yr** | **<11yr** |  |
| Neurodisability | 1.49% | 2.39% | 3.56% | - **0.2%** of children in Ontario had neurologic impairment associated with significant functional status limitations recorded in hospital admission records (subset of children with medical complexity)^1^ - **8.3%** of children **aged 6-10 years** in British Columbia were identified as having neurodevelopmental disorders and disabilities in primary or care or hospital admissions records)^19^ - **12%** of 2488 surveyed Canadian children aged  **10-11 years** in 1994/5 (including children classed as having neurodisability using scores on speech, mobility, cognition or dexterity from the Health Utilities Index)^20^ |
| **Neurodevelopmental conditions** | | | | |
| Autistic spectrum disorders (ASD) | 0.001% | 0.11% | 0.57% | - **1.5%** of 5–10-year-olds in England in 2017 (2.5% of boys, 0.4% of girls)^21^ - **1.8%** of children had ASD recorded as Special Educational Need (SEN) type of need in England between 2015-2022^22^ - **2.9%** of 10-14-year-olds registered with English primary care had a diagnosis of ASD^23^ |
| Learning disability | 0.003% | 0.04% | 0.13% | - **2.5%** of children in state funded schools in England in England in 2014/15 had a learning disability (defined as school-record of moderate learning difficulty (MLD), severe learning difficulty (SLD) and profound multiple learning difficulty (PMLD) as a type of SEN))^24^ - **0.1%** of children had PMLD and **0.4%** had SLD recorded as primary SEN type of need in England between 2015-2022^22^ - **2.5%** of children in April 2017 and March 2019 who were living in patient capture area for Sunderland Royal Hospital in England (capturing Sunderland, Washington, Houghton-Le-Spring, and Easington)^25^ |
| Developmental disorder | 0.014% | 0.34% | 0.75% |  |
| Hyperkinetic disorders | <10 children in the cohort | 0.01% | 0.27% | - **1.6%** of 5–10-year-olds in England in 2017 (2.6% boys & 0.6% girls)^21^ - **1.0%** of children in primary, secondary, or special schools in Scotland in 2008-9-2013-14 (*N* = 766,244) received medication for attention deficit hyperactivity disorder (ADHD)^26^ |
| Tic disorders | 0.0004% | 0.003% | 0.03% | - **0.6%** of 5–19-year-olds in England in 2017 had tic disorders^21^ |
| Behavioural (or ‘conduct’) disorders | 0.0004% | 0.02% | 0.07% | - **5.0%** of 5–10-year-olds in England in 2017 had a behavioural disorder^21^ |
| **Complex neurologic conditions** | | | | |
| Cerebral palsy (CP) | 0.03% | 0.20% | 0.28% | - **0.3%** (2.5-3.4 per 1000) children and young people aged 0–25 years old between 1 January 2004 and 31 December 2014 in England had cerebral palsy diagnosis in primary or secondary healthcare records^14^ - **0.3%** of children and young people aged 0-24 years old in Northern Ireland (captured in The Northern Ireland Cerebral Palsy Register)^27^ - **0.22%:** Sex-standardised period prevalence (based on data from primary and secondary healthcare in England) from 1 April 2010 to 31 Mar 2015 in children aged <10 years old was 22 per 10,000^28^ |
| Epilepsy | 0.10% | 0.32% | 0.52% | - **0.7%** of children in primary, secondary, or special schools in Scotland in 2008-9-2013-14 (*N* = 766,244) received antiepileptic medication^29^ - **0.4% to 0.7%** of children in England had epilepsy indicated in primary care records by age 5^30^ - Prevalence of epilepsy recorded in primary care records was between **0.1%-0.2% for children aged <5 years old** (1.07/1000 in CPRD Gold and 1.78/1000 in CPRD Aurum) and **0.3-0.5% in children aged 5-9 years old** (3.01/1000 in CPRD Gold and 4.91/1000 in CPRD Aurum)^31^ - **0.6%**: Sex-standardised period prevalence (based on data from primary and secondary healthcare in England) from 1 April 2010 to 31 Mar 2015 in children aged <10 years old was 61 per 10,000^28^ |
| **Congenital or inherited conditions** | | | | |
| **Anomalies of brain and central nervous system** | | | | |
| Anomalies of nervous system | 0.16% | 0.27% | 0.33% | - **0.09% of live births** in England in 2019 had anomalies of nervous system (registered with National Congenital Anomaly and Rare Disease Registration Service, NCARDRS)^32^ - **0.13% of live births** in 2003-2009 in European countries participating in EUROCAT (European network of population-based registries for the epidemiological surveillance of congenital anomalies) had anomalies of nervous system^33^ |
| Anencephaly | 0.001% | 0.001% | 0.001% | - **0.003%** of live births in England in 2019 (registered with NCARDRS)^32^ - **0.003%** of live births in 2003-2009 in European countries participating in EUROCAT^33^ |
| Encephalocele | 0.0046% | 0.0055% | 0.0062% | - **0.0038%** of live births in England in 2019 (registered with NCARDRS)^32^ - **0.0036%** of live births in 2003-2009 in European countries participating in EUROCAT^33^ |
| Microcephaly | 0.036% | 0.098% | 0.13% | - **0.008%** of live births in England in 2019 had severe microcephaly (registered with NCARDRS)^32^ - **0.025%** of live births in 2003-2009 in European countries participating in EUROCAT^33^ - **0.0055%** of live-born infants aged ≤12 months born in 2017-2018 were newly diagnosed with severe microcephaly (head circumference < (–3 standard deviations)^34^ |
| Spina bifida | 0.018% | 0.023% | 0.028% | - **0.021%** of live births in England in 2019 (registered with NCARDRS)^32^ - **0.019%** of live births in 2003-2009 in European countries participating in EUROCAT^33^ |
| Congenital hydrocephalus | 0.036% | 0.046% | 0.050% | - **0.027%** of live born children in England in 2019 (registered with NCARDRS)^32^ - **0.032%** of live births in 2003-2009 in European countries participating in EUROCAT^33^ |
| **Chromosomal anomalies** | | | | |
| Down | 0.10% | 0.11% | 0.11% | - **0.12%** in data linkage between HES and Down Syndrome registry^35^ - **0.11%** of live born children in England in 2019 (registered with NCARDRS)^32^ - **0.10%** of live births in 2003-2009 in European countries participating in EUROCAT^33^ |
| Edwards | 0.007% | 0.008% | 0.008% | - **0.009%** of live born children in England in 2019 (registered with NCARDRS)^32^ - **0.008%** of live births in 2003-2009 in European countries participating in EUROCAT^33^ |
| Patau | 0.004% | 0.004% | 0.004% | - **0.003%** of live born children in England in 2019 (registered with NCARDRS)^32^ - **0.004%** of live births in 2003-2009 in European countries participating in EUROCAT^33^ |
| Klinefelter’s syndrome | 0.003% | 0.004% | 0.006% | - **0.003%** of live born children in England in 2019 (registered with NCARDRS)^32^ |
| Fetal Alcohol Syndrome | 0.004% | 0.007% | 0.010% | - **0.0007%** of live born children in England in 2019 had FAS (registered with NCARDARS congenital anomaly registry)^32^ - **0.0034%** - incidence rate of 3.4/100 000 children aged 0–16 years in 2018-2019 in the UK and Ireland^36^ - **3.24%** (32.4 cases per 1,000 population) of children and young people in the United Kingdom had *fetal alcohol spectrum disorder (FASD)* according to a meta-analysis of global prevalence of FASD. Note that FASD includes a broader range of conditions, of which FAS is the most severe.^37^ |
| **Other conditions affecting brain** | | | | |
| Tumours of brain/central nervous system (CNS) | 0.005% | 0.018% | 0.046% | - **0.03%** (247 of 766,217) pupils attending state school between 2009 and 2013 in Scotland had a previous diagnosis of malignant neoplasms of eye, brain, or other parts of central nervous system^38^ - Crude prevalence of brain and other CNS tumours in children and adolescents aged 0-14 years old in the United States between 2014 and 2018 was **0.04%** (39.22/100,000).^39^ - **0.03%:** Sex-standardised period prevalence of primary malignancy in the brain (based on data from primary and secondary healthcare in England) from 1 April 2010 to 31 Mar 2015 in children aged <10 years old in England was 3 per 10,000^28^ |
| Hydrocephalus | 0.055% | 0.081% | 0.10% | - **0.16%** (164/100,000) of children in Europe had hydrocephalus according to meta-analysis of global estimates of hydrocephalus^40^ - **0.11%** (2,572 out of 2,381,413) live-born children in Denmark in 1997-2015 had infantile hydrocephalus (diagnosed before the age of 2 years old, including congenital hydrocephalus)^41^ |
| Paediatric stroke | 0.026% | 0.046% | 0.066% | - **Incidence** of paediatric ischemic stroke was **0.03%** (38.57 per 100,000 live births) **in neonates** and 0.001% (1.02/100,000, 0.67–1.56) in older children (according to a meta-analysis of incidence)^42^ - based on data from primary and secondary healthcare in England between 1 April 2010 to 31 March 2015, sex-standardised period prevalence in children aged <10 years old in England was 3 per 10,000 for ischaemic stroke and 3 per 10,000 for Intracerebral Haemorrhage^28^ |
| encephalitis | 0.005% | 0.016% | 0.023% | - **0.02%:** based on data from primary and secondary healthcare in England between 1 April 2010 to 31 March 2015, sex-standardised period prevalence in children aged <10 years old was 2 per 10,000^28^ |
| meningitis | 0.11% | 0.15% | 0.17% | - **0.15%:** sex-standardised period prevalence in children aged <10 years old based on data from primary and secondary healthcare in England between 1 April 2010 to 31 March 2015 was 15 per 10,000^28^ |
| Motor / movement disorders | | | | |
| Motor/movement disorders | 0.04% | 0.12% | 0.20% |  |
| spinal muscular atrophy | 0.007% | 0.012% | 0.015% | - Birth prevalence of 6.2 per 100,000 births between 2008 and 2016 in England (estimate based on the same hospital admission data)^43^ |
| Sensory impairment | | | | |
| Hearing Impairment | 0.02% | 0.11% | 0.23% | - On average, **0.27%** of all school-aged children had hearing impairment recorded as SEN type of need in England per school year between 2015-2022^22^ - based on data from primary and secondary healthcare in England between 1 April 2010 to 31 March 2015, sex-standardised period prevalence of deafness in children aged <10 years old was 301 per 10,000^28^ - prevalence of permanent bilateral severe to profound hearing loss detected through universal newborn hearing screening is 1.1 per 1000 newborns^44^ |
| Bilateral hearing impairment | 0.007% | 0.04% | 0.06% |  |
| Hearing aid (Cochlear or other) | 0.008% | 0.07% | 0.17% |  |
| Visual Impairment (VI, including conditions associated with high risk of VI) | 0.09% | 0.13% | 0.18% | - On average, **0.16%** of all school-aged children had visual impairment recorded as SEN type of need in England per school year between 2015-2022^22^ - **0.18%:** sex-standardised period prevalence of blindness in children aged <10 years old based on data from primary and secondary healthcare in England between 1 April 2010 to 31 March 2015 was 18 per 10,000^28^ - Cumulative incidence of bilateral VI from UK-wide cross-sectional observational study (BCVIS2) of children aged <18 years old newly diagnosed with VI in 2015-16 was:^45^   - **0.05%** (5.2 per 10 000 children) aged <1 years old   - **0.09%** (8.9 per 10 000 children) aged <5 years old   - **0.08%** (7.7 per 10 000 children) aged <15 years old |
| Bilateral visual impairment | 0.003% | 0.016% | 0.03% |  |
| Perinatal conditions | | | | |
| Severe birth asphyxia | 0.21% |  |  | - 2.6 per 1000 live births in 2015 had hypoxic-ischaemic encephalopathy (using NNRD)^46^ |
| Neonatal abstinence syndrome | 0.063% |  |  | - Incidence was 1.6/1000 live births in infants born between 2012 and 2017 who were admitted to a neonatal unit in England (using data from National Neonatal Research Database, NNRD)^47^ |
| Perinatal brain damage | 0.39% |  |  | - 4.90 per 1000 live births in 2012 and 5.19 per 1000 live births in 2015 (using NNRD)^46^ |
| Perinatal seizures | 0.18% |  |  | - Between 2.1 per 1000 live births in 2012 and 1.9 per 1000 live births in 2015 (using NNRD)^46^ |
| Intracranial   haemorrhage | 0.12% |  |  | - 1.1 per 1000 live births in 2015 (using NNRD)^46^ |
| Hypoxic-ischaemic   encephalopathy | 0.0006% |  |  | - 2.6 per 1000 live births in 2015 (using NNRD)^46^ |
| CNS infection | 0.044% |  |  | - Between 0.51 per 1000 live births in 2012 and 0.70 (0.64 to 0.77) per 1000 live births in 2015 (using NNRD)^46^ |
| Perinatal stroke | 0.07% |  |  | - **0.014%**: 0.14 (0.11 to 0.17) per 1000 live births in 2015 (using data on NICU admissions from National Neonatal Research Database, NNRD)^46^ - **Incidence** of paediatric ischemic stroke was **0.03%** (38.57 per 100,000 live births) **in neonates** (according to a meta-analysis of incidence)^42^ |
| Congenital infections | 0.023% | 0.024% | 0.024% |  |
| Cytomegalovirus | 0.010% | 0.010% | 0.011% | - 0.014% - 14 per 100,000 infants in 2016 were hospitalised due to cytomegalovirus^48^ |
| Rubella | <10 cases (<0.00028%) | | | - 0.00023%; in 2003–16 there were 31 rubella infections in pregnancy identified through routine surveillance in the UK (0.23 per 100,000 pregnancies)^49^ - 0.0002% - 0.2 per 100,000 infants in 2016 were hospitalised due to congenital rubella^48^ |

Appendix Figure 4 - Proportion of boys by specific neurodisability conditions

CNS: central nervous system, HI: hearing impairment, VI: visual impairment
*calculated as % of children with no missing data.

Appendix Figure 5 – Proportion of children by gestational age category (including missing data) and by specific neurodisability conditions CNS: central nervous system

Appendix Figure 6 – Proportion of children who died aged <11 years old by specific neurodisability conditions

*CNS: central nervous system*

Appendix Table 5 – Proportion of children according to school-recorded types of need for Special Educational Needs (SEN) provision (as a proportion of children with any SEN provision)

|  | Condition | **Number with any SEN provision** | **% with speech, language & communication** | **% with moderate learning difficulty** | **% with earning disability** | **% with Autistic Spectrum Disorders** | **% with Hearing impairment** | **% with visual impairment** | **% with physical disability** |
| --- | --- | --- | --- | --- | --- | --- | --- | --- | --- |
|  | Any neurodisability | 83,586 | 30 | 25 | 18 | 21 | 7 | 5 | 16 |
| **Neuro-developmental conditions** | Learning disability | 4,239 | 31 | 23 | 52 | 18 | 6 | 11 | 24 |
|  | Developmental Disorders | 24,141 | 41 | 27 | 34 | 19 | 6 | 7 | 19 |
|  | Autism | 19,270 | 31 | 16 | 20 | 71 | 2 | 2 | 6 |
|  | Hyperkinetic disorders | 8,980 | 28 | 26 | 10 | 28 | 2 | 2 | 6 |
|  | Behavioural disorders | 2,268 | 28 | 27 | 18 | 20 | 3 | 4 | 13 |
|  | Tic disorders | 652 | 24 | 21 | 7 | 21 | 3 | 4 | 10 |
|  | Cerebral Palsy | 8,499 | 18 | 16 | 35 | 5 | 5 | 11 | 61 |
|  | Epilepsy | 13,227 | 25 | 24 | 34 | 13 | 4 | 8 | 22 |
| **congenital/inherited conditions** | Chromosomal anomalies | 5,462 | 35 | 26 | 59 | 7 | 8 | 6 | 17 |
|  | Down Syndrome | 3,398 | 36 | 27 | 66 | 4 | 8 | 5 | 12 |
|  | Anomalies of nervous system | 8,305 | 26 | 23 | 39 | 8 | 6 | 12 | 31 |
|  | Microcephaly | 3,792 | 30 | 24 | 52 | 8 | 6 | 13 | 23 |
|  | Congenital hydrocephalus | 1,209 | 22 | 22 | 37 | 6 | 6 | 13 | 40 |
|  | Spina Bifida | 689 | 15 | 17 | 14 | 3 | 4 | 3 | 61 |
|  | Foetal Alcohol Syndrome | 276 | 33 | 39 | 20 | 7 | 7 | 8 | 18 |
|  | Sex chromosome anomalies | 1,917 | 32 | 23 | 46 | 12 | 7 | 8 | 24 |
|  | Metabolic | 2,073 | 21 | 21 | 27 | 5 | 7 | 16 | 24 |
| **Other conditions of brain** | Hydrocephalus | 2,497 | 21 | 22 | 28 | 6 | 6 | 11 | 39 |
|  | Stroke | 1,508 | 24 | 23 | 27 | 6 | 5 | 10 | 43 |
|  | Tumours | 915 | 17 | 25 | 12 | 7 | 5 | 11 | 26 |
|  | Inflammatory | 3,911 | 23 | 27 | 13 | 7 | 8 | 5 | 13 |
|  | Encephalitis | 384 | 32 | 24 | 19 | 9 | * | 6 | 14 |
|  | Meningitis | 2,577 | 21 | 29 | 9 | 7 | 10 | 4 | 9 |
| **Sensory impairment** | Hearing Impairment | 6,077 | 27 | 15 | 17 | 6 | 56 | 6 | 11 |
|  | Bilateral hearing impairment | 1,977 | 22 | 9 | 16 | 4 | 80 | 7 | 10 |
|  | Hearing aid (Cochlear or other) | 4,352 | 27 | 16 | 15 | 6 | 53 | 6 | 10 |
|  | Visual Impairment | 4,721 | 19 | 19 | 38 | 6 | 9 | 32 | 22 |
|  | Bilateral visual impairment | 947 | 6 | 5 | 69 | 4 | 10 | 54 | 21 |
|  | Retinopathy of prematurity | 1,743 | 29 | 31 | 17 | 7 | 11 | 12 | 20 |
| **Motor Function Impairment** | Any Motor Function Impairment | 4,789 | 20 | 17 | 33 | 6 | 5 | 10 | 45 |
|  | Central nervous system (CNS) Degenerative conditions | 550 | 22 | 16 | 26 | 3 | 7 | 8 | 60 |
|  | Spinal Muscular Dystrophy | 284 | 20 | 11 | 31 | * | 6 | 9 | 66 |
|  | Myoneural | 1,381 | 21 | 18 | 29 | 5 | 4 | 8 | 58 |
|  | Movement disorders | 1,465 | 17 | 13 | 49 | 7 | 5 | 12 | 39 |
|  | Polyneuropathies | 440 | 18 | 18 | 17 | 3 | 6 | 7 | 42 |
| **Perinatal conditions** | Severe birth asphyxia | 2,453 | 25 | 24 | 11 | 7 | 4 | 2 | 14 |
|  | Perinatal brain injury | 6,496 | 25 | 25 | 23 | 7 | 6 | 8 | 29 |
|  | Perinatal seizures | 2,976 | 26 | 24 | 26 | 7 | 6 | 8 | 25 |
|  | Perinatal Stroke | 1,095 | 27 | 25 | 24 | 6 | 6 | 8 | 33 |
|  | Intracranial Haemorrhage | 2,074 | 24 | 27 | 21 | 6 | 7 | 9 | 31 |
|  | CNS Infection | 639 | 23 | 30 | 18 | 7 | 5 | 6 | 15 |
|  | Neonatal Abstinence Syndrome | 1,087 | 20 | 30 | 3 | 5 | 2 | 2 | 4 |
|  | Congenital infections | 403 | 27 | 22 | 22 | 7 | 17 | 9 | 14 |
|  | extremely low birthweight | 3,442 | 29 | 33 | 11 | 7 | 7 | 6 | 15 |
|  | Extreme preterm birth | 1,855 | 30 | 32 | 13 | 8 | 9 | 8 | 17 |

* indicates supressed calculation due to underlying counts being <10

# References

1 Cohen E, Berry JG, Camacho X, Anderson G, Wodchis W, Guttmann A. Patterns and costs of health care use of children with medical complexity. *Pediatrics* 2012; **130**: e1463-1470.

2 Thomson JE, Feinstein JA, Hall M, Gay JC, Butts B, Berry JG. Identification of Children With High-Intensity Neurological Impairment. *JAMA Pediatrics* 2019; **173**: 989–91.

3 Berry JG, Poduri A, Bonkowsky JL, *et al.* Trends in Resource Utilization by Children with Neurological Impairment in the United States Inpatient Health Care System: A Repeat Cross-Sectional Study. *PLOS Medicine* 2012; **9**: e1001158.

4 Hardelid P, Dattani N, Gilbert R. Estimating the prevalence of chronic conditions in children who die in England, Scotland and Wales: a data linkage cohort study. *BMJ Open* 2014; **4**: e005331.

5 Feudtner C, Feinstein JA, Zhong W, Hall M, Dai D. Pediatric complex chronic conditions classification system version 2: updated for ICD-10 and complex medical technology dependence and transplantation. *BMC Pediatrics* 2014; **14**: 199.

6 Fraser LK, Gibson-Smith D, Jarvis S, Norman P, Parslow RC. Estimating the current and future prevalence of life-limiting conditions in children in England. *Palliat Med* 2021; **35**: 1641–51.

7 Jarvis SW, Livingston J, Childs A-M, Fraser L. The impact of neurological disorders on hospital admissions for children and young people: a routine health data study. *Int J Popul Data Sci*; **3**: 421.

8 Sheehan R, Mansour H, Broadbent M, *et al.* Recording of intellectual disability in general hospitals in England 2006–2019: Cohort study using linked datasets. *PLOS Medicine* 2023; **20**: e1004117.

9 Zylbersztejn A, Stilwell PA, Zhu H, *et al.* Trends in hospital admissions during transition from paediatric to adult services for young people with learning disabilities or autism: population-based cohort study. *The Lancet Regional Health – Europe* 2023; **24**. DOI:10.1016/j.lanepe.2022.100531.

10 Gale NK, Heath G, Cameron E, Rashid S, Redwood S. Using the framework method for the analysis of qualitative data in multi-disciplinary health research. *BMC Medical Research Methodology* 2013; **13**: 117.

11 Knight HE, Oddie SJ, Harron KL, *et al.* Establishing a composite neonatal adverse outcome indicator using English hospital administrative data. *Arch Dis Child Fetal Neonatal Ed* 2019; **104**: F502–9.

12 Barsch Bergqvist A, Simatou E, Skiöld B, Mitha A, Bolk J. Term-born infants with a perinatal stroke frequently had seizures and were prescribed anti-seizure medication at discharge. *Acta Paediatrica* 2023; **112**: 1907–15.

13 Milan JB, Jensen TSR, Nørager N, *et al.* The ASPECT Hydrocephalus System: a non-hierarchical descriptive system for clinical use. *Acta Neurochir* 2022; published online Nov 24. DOI:10.1007/s00701-022-05412-6.

14 Carter B, Verity Bennett C, Bethel J, Jones HM, Wang T, Kemp A. Identifying cerebral palsy from routinely-collected data in England and Wales. *Clin Epidemiol* 2019; **11**: 457–68.

15 Guttmann A, Blackburn R, Amartey A, *et al.* Long-term mortality in mothers of infants with neonatal abstinence syndrome: A population-based parallel-cohort study in England and Ontario, Canada. *PLOS Medicine* 2019; **16**: e1002974.

16 Zylbersztejn A, Gilbert R, Hardelid P. Developing a national birth cohort for child health research using a hospital admissions database in England: The impact of changes to data collection practices. *PLOS ONE* 2020; **15**: e0243843.

17 Office for National Statistics. Birth statistics, England and Wales (Series FM1). Office for National Statistics. https://webarchive.nationalarchives.gov.uk/ukgwa/20160108035551mp_/http://www.ons.gov.uk/ons/rel/vsob1/birth-statistics--england-and-wales--series-fm1-/index.html (accessed Nov 25, 2024).

18 Office for National Statistics. Births in England and Wales: summary tables. https://www.ons.gov.uk/peoplepopulationandcommunity/birthsdeathsandmarriages/livebirths/datasets/birthsummarytables (accessed Nov 25, 2024).

19 Arim RG, Miller AR, Guèvremont A, Lach LM, Brehaut JC, Kohen DE. Children with neurodevelopmental disorders and disabilities: a population-based study of healthcare service utilization using administrative data. *Developmental Medicine & Child Neurology* 2017; **59**: 1284–90.

20 Sentenac M, Lach LM, Gariepy G, Elgar FJ. Education disparities in young people with and without neurodisabilities. *Developmental Medicine & Child Neurology* 2019; **61**: 226–31.

21 Mental Health of Children and Young People in England, 2017 [PAS]. NHS England Digital. https://digital.nhs.uk/data-and-information/publications/statistical/mental-health-of-children-and-young-people-in-england/2017/2017 (accessed Aug 20, 2024).

22 Department for Education. Year group, by type of SEN provision and type of need - 2016 to 2023’ from ’Special educational needs in England. https://explore-education-statistics.service.gov.uk/data-tables/permalink/0706944f-3e40-4815-fd40-08dc6b74a79d (accessed May 5, 2024).

23 O’Nions E, Petersen I, Buckman JEJ, *et al.* Autism in England: assessing underdiagnosis in a population-based cohort study of prospectively collected primary care data. *The Lancet Regional Health - Europe* 2023; **29**: 100626.

24 Public Health England. People with learning disabilities in England 2015: Main report. Learning Disabilities Observatory. 2016. https://assets.publishing.service.gov.uk/media/5a81e329ed915d74e3400976/PWLDIE_2015_main_report_NB090517.pdf.

25 Horridge KA, Bretnall G, Fraser LK. Hospital admissions of school-age children with an intellectual disability: A population-based survey. *Developmental Medicine & Child Neurology* 2023; **65**: 1511–9.

26 Fleming M, Bandyopadhyay A, McLay JS, *et al.* Age within schoolyear and attention-deficit hyperactivity disorder in Scotland and Wales. *BMC Public Health* 2022; **22**: 1070.

27 Carter B, Bennett CV, Jones H, *et al.* Healthcare use by children and young adults with cerebral palsy. *Developmental Medicine & Child Neurology* 2021; **63**: 75–80.

28 Kuan V, Denaxas S, Gonzalez-Izquierdo A, *et al.* A chronological map of 308 physical and mental health conditions from 4 million individuals in the English National Health Service. *The Lancet Digital Health* 2019; **1**: e63–77.

29 Fleming M, Fitton CA, Steiner MFC, *et al.* Educational and health outcomes of children and adolescents receiving antiepileptic medication: Scotland-wide record linkage study of 766 244 schoolchildren. *BMC Public Health* 2019; **19**: 595.

30 Meeraus WH, Petersen I, Chin RF, Knott F, Gilbert R. Childhood epilepsy recorded in primary care in the UK. *Archives of Disease in Childhood* 2013; **98**: 195–202.

31 Wigglesworth S, Neligan A, Dickson J, *et al.* The incidence and prevalence of epilepsy in the United Kingdom 2013–2018: A retrospective cohort study of UK primary care data. *Seizure: European Journal of Epilepsy* 2023; **105**: 37–42.

32 Public Health England. NCARDRS congenital anomaly statistics: annual data. GOV.UK. 2021; published online Sept 29. https://www.gov.uk/government/publications/ncardrs-congenital-anomaly-annual-data (accessed Aug 20, 2024).

33 European Platform on Rare Disease Registration. EUROCAT Data: Prevalence charts and tables. https://eu-rd-platform.jrc.ec.europa.eu/eurocat/eurocat-data/prevalence_en (accessed Oct 30, 2023).

34 Knowles RL, Solebo AL, Sampaio MA, *et al.* Incidence, aetiology and neurodisability associated with severe microcephaly: a national surveillance study. *Archives of Disease in Childhood* 2023; **108**: 211–7.

35 Doidge JC, Morris JK, Harron KL, Stevens S, Gilbert R. Prevalence of Down’s Syndrome in England, 1998–2013: Comparison of linked surveillance data and electronic health records. *International Journal of Population Data Science* 2020; **5**. DOI:10.23889/ijpds.v5i1.1157.

36 Burleigh CR, Lynn RM, Verity C, Winstone AM, White SR, Johnson K. Fetal alcohol syndrome in the UK. *Archives of Disease in Childhood* 2023; **108**: 852–6.

37 Lange S, Probst C, Gmel G, Rehm J, Burd L, Popova S. Global Prevalence of Fetal Alcohol Spectrum Disorder Among Children and Youth: A Systematic Review and Meta-analysis. *JAMA Pediatr* 2017; **171**: 948–56.

38 Baughan N, Pell JP, Mackay DF, Clark D, King A, Fleming M. Educational outcomes in childhood cancer survivors: A Scotland-wide record-linkage study of 766,217 schoolchildren. *PLOS ONE* 2023; **18**: e0286840.

39 Ostrom QT, Price M, Ryan K, *et al.* CBTRUS Statistical Report: Pediatric Brain Tumor Foundation Childhood and Adolescent Primary Brain and Other Central Nervous System Tumors Diagnosed in the United States in 2014–2018. *Neuro Oncol* 2022; **24**: iii1–38.

40 Isaacs AM, Riva-Cambrin J, Yavin D, *et al.* Age-specific global epidemiology of hydrocephalus: Systematic review, metanalysis and global birth surveillance. *PLOS ONE* 2018; **13**: e0204926.

41 Schmidt LB, Corn G, Wohlfahrt J, Melbye M, Munch TN. School performance in children with infantile hydrocephalus: a nationwide cohort study. *Clinical Epidemiology* 2018; **10**: 1721–31.

42 Gao L, Lim M, Nguyen D, *et al.* The incidence of pediatric ischemic stroke: A systematic review and meta-analysis. *International Journal of Stroke* 2023; **18**: 765–72.

43 Spinal muscular atrophy type 1: NCARDRS report. NDRS. https://digital.nhs.uk/ndrs/data/data-outputs/spinal-muscular-atrophy-type-1-ncardrs-data-briefing/spinal-muscular-atrophy-type-1-ncardrs-report (accessed Nov 28, 2024).

44 Butcher E, Dezateux C, Cortina-Borja M, Knowles RL. Prevalence of permanent childhood hearing loss detected at the universal newborn hearing screen: Systematic review and meta-analysis. *PLOS ONE* 2019; **14**: e0219600.

45 Teoh LJ, Solebo AL, Rahi JS, *et al.* Visual impairment, severe visual impairment, and blindness in children in Britain (BCVIS2): a national observational study. *The Lancet Child & Adolescent Health* 2021; **5**: 190–200.

46 Gale C, Statnikov Y, Jawad S, Uthaya SN, Modi N. Neonatal brain injuries in England: population-based incidence derived from routinely recorded clinical data held in the National Neonatal Research Database. *Arch Dis Child Fetal Neonatal Ed* 2018; **103**: F301–6.

47 Rees P, Carter B, Gale C, Petrou S, Botting B, Sutcliffe AG. Cost of neonatal abstinence syndrome: an economic analysis of English national data held in the National Neonatal Research Database. *Arch Dis Child Fetal Neonatal Ed* 2021; **106**: 494–500.

48 Kadambari S, Pollard AJ, Goldacre MJ, Goldacre R. Congenital viral infections in England over five decades: a population-based observational study. *The Lancet Infectious Diseases* 2020; **20**: 220–9.

49 Bukasa A, Campbell H, Brown K, *et al.* Rubella infection in pregnancy and congenital rubella in United Kingdom, 2003 to 2016. *Euro Surveill* 2018; **23**: 17–00381.
